# The Roles of Health and e-Health Literacy, Conspiracy Beliefs and Political Sympathy in the Adherence to Preventive Measures Recommended during the Pandemic

**DOI:** 10.3390/ijerph19148346

**Published:** 2022-07-08

**Authors:** Mariusz Duplaga

**Affiliations:** Department of Health Promotion and e-Health, Institute of Public Health, Faculty of Health Sciences, Jagiellonian University Medical College, 31-066 Kraków, Poland; mariusz.duplaga@uj.edu.pl

**Keywords:** COVID-19, preventive measures, health literacy, e-health literacy, conspiracy beliefs, conspiracy theories, political sympathy, religious practices

## Abstract

Adherence to preventive measures is of the utmost importance for limiting the spreading of the coronavirus. Many predictors of adopting preventive behaviors have been analyzed in different countries during the COVID-19 pandemic. Objectives: The study’s main aim was to assess the roles of health (HL) and e-health literacy (eHL), conspiracy beliefs, political sympathy, and religious practices in the adherence to COVID-19 preventive measures after adjusting for sociodemographic factors. The analysis was based on the data obtained from a survey conducted among 2410 adult Internet users in Poland in October 2020. The survey was performed with a computer-assisted web-based interviewing technique. Survey data were analyzed through uni- and multivariable linear regression models. The multivariable regression model revealed that after adjusting for sociodemographic variables, the preventive measures score (PMS) was significantly associated with HL (B = 0.04, *p* < 0.001), eHL (B = 0.03, *p* < 0.001) and the COVID-19-related conspiracy beliefs score (C19CBS) (B = −0.24, *p* < 0.001). There was also a significant statistical relationship between PMS and political sympathies and religious practices. HL and eHL are key factors eligible for modification through appropriate interventions and showing positive effects in compliance with preventive measures. Conspiracy beliefs, political sympathy and religious beliefs are independently associated with the adherence.

## 1. Introduction

The COVID-19 pandemic quickly became a great challenge to modern public health systems. In the first phase of the pandemic, before vaccines against COVID-19 became available, the main efforts of governments were focused on preventive measures, limiting the transmission of the new coronavirus. Recommendations distributed to citizens usually included social distancing, wearing masks, and hand hygiene care. Other measures were also emphasized depending on the country, e.g., covering the nose and mouth when sneezing and coughing or avoiding touching the face with hands. The successful execution of these preventive measures largely depended on the compliance of societies with the recommended procedures.

From the beginning of the pandemic, health (HL) and e-health literacy (eHL) were underlined as key determinants of the implementation of COVID-19 preventive measures [1,2,3,4]. HL is usually defined as the cognitive and social skills shaping the motivation and ability of people to access, understand and apply the information for promoting and maintaining good health [5]. In turn, eHL is perceived as a set of skills that enables accessing, understanding, appraising and using the information available from electronic sources, mainly the Internet, for the same purposes [6]. In the context of the COVID-19 pandemic, both HL and eHL are perceived as determinants of efficient access to reliable information about COVID-19 infection and protection against the flood of accompanying misinformation [7,8].

The belief in fast-spreading conspiracy theories was also indicated as a possible factor of non-compliance [9,10,11]. Some authors showed that political partisanship might play a role in some countries [12,13,14], especially if it was combined with extreme political views or the support for political leaders who sought popularity by spreading health denialism [9].

Among other factors, the perceived risk from COVID-19 [15,16,17], trust in science [9], trust in the government’s competence [10,18,19,20,21], positive attitudes toward complying with recommendations [14], intellectual curiosity [9], low impulsivity [15] and high empathy [21] were indicated as features favoring the adherence to recommended preventive actions.

Among sociodemographic variables, gender [17,20,22,23], age [17,21], place of residence [24,25], the level of education [25], vocational status [20,25], socioeconomic status [17], marital status [20] and having children [20] were reported to be significantly associated with the adoption of preventive measures. Most studies revealed that women, persons with a higher level of education and older adults are more likely to adhere to COVID-19 preventive measures than males, persons with lower education and younger persons [17,21,22,25]. However, some authors also found that sociodemographic factors may have an insignificant or low impact on COVID-19 preventive behaviors compared to such variables as vulnerability or perceived risk [16,23].

The study’s main aim was to analyze the determinants of adherence to COVID-19 preventive measures in the Polish population of adult Internet users in the pre-vaccine period of the pandemic. The roles of HL and eHL, conspiracy beliefs related to the emergence of the COVID-19 pandemic, political sympathies and devotion to religious practices were assessed after adjusting for sociodemographic variables. The hypotheses formulated based on the prior studies are as follows: (1) higher levels of HL and eHL are associated with greater adherence to preventive measures; (2) conspiracy beliefs are associated with lower adherence to preventive measures; (3) right-wing political sympathies are significantly associated with lower adherence; (4) higher involvement in religious practices is associated with higher adherence.

## 2. Materials and Methods

### 2.1. Survey

The analysis presented in this paper is based on the data from a computer-assisted web-based interviewing (CAWI) survey performed among a sample of Polish adult Internet users. The survey was carried out in October 2020 at the beginning of the second wave of the COVID-19 pandemic in Poland. The study sample, including 2410 respondents, was adjusted according to gender, age, level of education, place of residence, and NUTS1 region, in line with the data obtained for adult Internet users by Statistics Poland, the national statistical office [26]. Assuming a population size of 28,600,000 [26], a confidence level of 0.95, and a fraction of 0.5, the sampling error given the size of the study sample is equal to 2.0%. The survey was conducted by BIOSTAT, a poll opinion company selected following obligatory university tender procedures.

Before joining the study, the respondents had to provide informed consent in line with the procedures of CAWI surveys performed by the company. The research team provided information about the survey as required by the Bioethical Committee at Jagiellonian University. The protocol for the study was accepted by the Bioethical Committee of the Jagiellonian University—decision No 1072.6120.99.2020 from 23 April 2020, with further amendments.

### 2.2. Questionnaire

The questionnaire used in the survey consisted of 66 items. The HL level of respondents was assessed using the 16-item European Health Literacy Survey Questionnaire (HLS-EU-Q16) [27]. eHL was measured on the Polish version of the e-health literacy Scale (Pl-eHEALS), originally developed by Norman and Skinner [28,29]. Conspiracy beliefs related to COVID-19 were assessed on an ad hoc scale consisting of 6 items based on a 7-point Likert scale. The questionnaire also included six items asking about adherence to the preventive measures recommended by the Polish Ministry of Health during the COVID-19 pandemic. Finally, a set of items was used to assess respondents’ sociodemographic and economic characteristics, political views and religious practices.

### 2.3. Variables

The score reflecting the adherence to preventive measures during the pandemic was established based on responses to six items asking about following the recommendations on social distance, wearing a mask, washing hands, avoiding crowded places, not touching eyes, nose and mouth with hands and covering the nose and mouth when sneezing or coughing. The responses to these items could be provided based on the 7-point frequency scale from ‘never’ (assigned ‘1’) to ‘always’ (assigned ‘7’). The preventive measures score (PMS) was calculated as a mean of the responses to individual items asking about six preventive measures, converted to numerical values. The internal consistency of this ad hoc scale remained at a good level (Cronbach’s = 0.869, Guttman half-split coefficient = 0.848).

The HL score was calculated based on responses to the HLS-EU-Q16 questionnaire in line with the recommendations of the research team that developed the scale [27]. The responses ‘easy’ and ‘very easy’ were coded as ‘1’, and ‘difficult’ and ‘very difficult’ as ‘0’. The response ‘difficult to say’ was treated as a missing value. The score was calculated as a sum of responses to 16 items only if the number of missing values did not exceed three.

The eHL score was obtained as a sum of the responses to eight items of the Pl-eHEALS scale after their conversion to numeric values from ‘1’ for ‘decidedly disagree’ to ‘5’ for ‘decidedly agree’ [29]. The score could range from 8 to 40.

The COVID-19-related conspiracy beliefs score (C19CBS) was established based on the six items asking about specific conspiracy theories popular in Polish society. The responses to these items could be provided according to a 7-point Likert scale from ‘definitely false’ (assigned value ‘1’) to ‘definitely true’ (value ‘7’). The score was calculated as a mean of the responses to these six items. The assessment of the internal reliability of this scale yielded acceptable results (Cronbach’s α = 0.804, Guttman half-split coefficient = 0.792)

Sociodemographic variables included in the analysis reflected gender, age (as a continuous variable), place of residence (6 options from ‘rural’ to ‘urban-at least 500,000 inhabitants’), level of education (4 options from ‘lower than secondary’ to ‘university Master’s’), marital status (4 options: ‘married’, ‘single’, ‘partnership’, ‘widowed/divorced/separated’), vocational status (5 options: ‘employee of public or private sector’, ‘self-employed or farmer’, ‘on disability pension or retired’, ‘high school or university student’, ‘vocationally passive including unemployed’), and finally, monthly net income per household member (4 options: ‘≤1000 Polish zloty (PLN)’, ‘1001–2000 PLN’, ‘2001–3000 PLN’, and ‘>3001 PLN’).

The variables reflecting the time spent on social media per day were also included in the analysis. This variable had five response options, from “not used” to “at least 120 min”. The items of the survey questionnaire used for the development of variables analyzed in regression models are presented in Supplementary File S1.

Political views were assessed based on responses to the item asking about voting decisions during the last parliamentary elections in 2019. The respondents could provide a response indicating the main political parties or not participating in the election. Finally, the item asking about participation in religious practices could assume five options: ‘non-believer’, ‘non-practicing believer’, ‘less than once a month’, ‘1–3 times per month’, and ‘at least four times a month’.

### 2.4. Statistical Analysis

The analysis of data from the survey was carried out with the IBM SPSS v.24 package (IBM Corp., Armonk, NY, USA). The categorical variables were expressed as absolute frequencies and percentages. For numerical variables, means and standard deviations were calculated.

The analysis of determinants of PMS was carried out with linear regression modeling. In the first stage, the univariate models for PMS as a dependent variable and relevant independent variables were developed, including HL, eHL, C19CBS, age, gender, place of residence, level of education, marital status, vocational status, income level, political views and religious practices. In the next stage, a multivariable linear regression model was developed, including as independent variables only those variables that were significantly associated with PMS in the univariable models. For independent variables included in the uni- and multivariable regression models, unstandardized regression coefficients (B), standard errors (SE), standardized regression coefficients (β), 95% confidence intervals (95% CI) and *p* values were provided. For the multivariable model, collinearity was assessed, and VIP and tolerance were calculated for the variables included in the model. *p*-values < 0.05 were deemed significant.

## 3. Results

### 3.1. Characteristics of the Study Group

The percentage of women in the study group was 51.2%, 34.5% were residents of rural areas, 28.2% had a university education, and 67.0% were married persons or living with a partner. The mean age (SD) was 40.84 (14.47); the mean HL was 12.15 (3.70); the mean eHL was 25.34 (4.54); and finally, the mean C19CBS was 3.78. Detailed characteristics of the study group are provided in Table 1.

### 3.2. Conspiracy Beliefs

A significant portion of the respondents believed in conspiracy theories (Table 2). As many as 45.31% of the respondents agreed that the pandemic is a pretext for achieving hidden economic and political purposes, 39.80% agreed that the new coronavirus was a result of genetic manipulations, and 39.50% agreed that it was purposefully released from the laboratory. Even the theory stating that 5G networks can be involved in spreading the pandemic was supported by a considerable number (13.20%) of respondents.

### 3.3. Preventive Measures

Only 26.0% of the respondents declared that they always adhere to the requirement of social distancing. In turn, as many as 6.5% never or rarely kept social distance. Masks were always used by 62.6% and nearly always by the next 15.1% of respondents. Hands were always washed after possible contact with contaminated material or other people by 47.5% and nearly always washed by the next 20.3% of respondents. Detailed information about the distribution of responses to specific items asking about adhering to the recommended preventive measures is shown in Table 3. Mean individual scores obtained after conversion of responses to numerical values ranged from 5.28 (1.72) for avoiding touching the face with the hands to 6.08 (1.52) for wearing masks (Table 2). The mean PMS in the study group was 5.54 (1.24).

### 3.4. Univariate Linear Regression Modeling

PMS was significantly associated with HL, eHL, C19CBS, age, gender, marital status, vocational status, income level, political sympathy and religious practices (Table 4). Older persons adhered more to preventive guidelines than younger persons (B = 0.01, *p* < 0.001). Respondents with higher level of HL and eHL achieved higher PMS (B = 0.06, *p* < 0.001, and B = 0.05, *p* < 0.001, respectively). A higher level of conspiracy beliefs was associated with lower compliance with preventive guidelines (B = −0.23, *p* < 0.001).

Males showed lower PMS than females (B = −0.32, *p* < 0.001). Persons from households with a net monthly income per household member of at least 3001 PLN were less likely to adhere to preventive measures than those with incomes of 1001–2000 PLN (B = −0.16, *p* = 0.020). Retired persons or those on a disability pension, as well as vocationally passive persons, were more likely to adhere to preventive measures than employees (B = 0.55, *p* < 0.001 and B = 0.20, *p* = 0.003, respectively). Single people and persons living in a partnership were less likely to follow preventive measures than married persons (B = −0.24, *p* < 0.001, and B = 0.18, *p* = 0.010, respectively).

Compliance with preventive measures was also significantly associated with political sympathy and religious practices. Respondents who voted for the extreme right-wing Confederation Party and those who did not participate in the 2019 parliamentary election were much less likely to adhere to recommendations than those who voted for the winning Law and Justice party (B = −0.71 *p* < 0.001 and B = −0.31, *p* < 0.001).

### 3.5. Multivariable Linear Regression

The multivariable regression model confirmed the associations observed in the univariable models for HL, eHL, C19CBS, age, gender, level of income, vocational status, political sympathy and religious practices (ANOVA F = 17,439, *p* < 0.001, R^2^ = 0.17, corrected R^2^ = 0.16). Detailed results of the multivariable analysis are shown in Table 5.

## 4. Discussion

Our analysis demonstrated that adherence to preventive measures recommended during the pandemic depends on many factors, not only the knowledge and skills usually covered by the concept of HL or eHL, but also beliefs in conspiracy theories, political sympathy, attitude to religion, sociodemographic and economic variables.

In general, the first three hypotheses formulated for this study were confirmed. The multivariable regression analysis confirmed that females (more than males), older respondents (more than younger) and those with higher levels of HL and eHL are more likely to comply with preventive measures. In turn, singles and widowed or divorced persons are less likely than married respondents to adhere to prevention. Persons on a disability pension, retired or vocationally passive observe preventive measures more than employees. The respondents from the group with the highest income are less prone to adhere to recommendations than persons from the group with the lowest income level. Extreme right-wing political views or abstaining from voting are associated with a lower level of adherence in comparison to the right-wing party, Peace and Justice, which won the elections in 2019. Only the hypothesis about a significant relationship between involvement in religious practices and adherence was not confirmed. Non-believers are less likely to follow preventive measures than non-practicing believers.

The role of HL as a key factor that can influence the effectiveness of preventive measures was signaled from the beginning of the COVID-19 pandemic [30]. In 2021, Araujo et al. called HL a weapon against the virus [31]. Recently, Okan et al. have even used the term ‘social vaccine’ in relation to HL in the context of the COVID-19 pandemic [32]. Indeed, many authors of the subsequent studies confirmed a significant association between HL and attitudes and knowledge about preventive measures during the COVID-19 pandemic [33,34,35,36] or compliance with preventive measures [35,37,38,39,40] in various populations. Interestingly, some authors reported that HL is associated with knowledge and positive attitudes toward COVID-19 preventive measures but not with adopting preventive behaviors [36,41].

Fukuda et al. showed that HL is a determinant of engaging in preventive measures among educators in the educational setting [42]. Wong et al. [38] showed that the association between HL and preventive behaviors was mediated by COVID-19 information sharing with family members. Another study in China revealed that this association is mediated by perceived barriers and benefits, self-efficacy, trust in doctors’ social media and trust in TV news [43].

Wang et al. proposed a conceptual model in which preventive behaviors during the pandemic depend on infectious disease-specific HL, which is, in turn, modeled by such factors as situational predictors, including the accessibility of health information resources and support from the employee’s companies, as well as personal predictors including such features as age, gender, marital status, education and occupation [44].

According to the systematic review prepared by Ameri et al., enhancing the level of eHL is treated as a key method of increasing adherence to the guidelines on preventive measures during the COVID-19 pandemic [45]. Li and Liu demonstrated that higher eHL predicted individual preventive behaviors for COVID-19 among Internet users in China [46].

The negative influence of conspiracy beliefs on adhering to preventive measures during the COVID-19 pandemic was reported by some authors. Banai et al. observed that conspiracy beliefs on COVID-19 were directly and indirectly mediated by trust in governmental officials and associated with lower adoption of preventive measures [10]. Farias and Pilati found that beliefs in conspiracy theories focusing on control of information predicted non-compliance with social distancing [11]. In contrast, no association between COVID-19-related conspiracy beliefs and preventive measures was reported for the Turkish population [15].

Sánchez-Arenas et al. also reported similar sociodemographic factors favoring the adoption of preventive measures as in our study for the general population in Mexico [40]. They observed that females, older persons and retired persons were more likely to demonstrate preventive behaviors.

The study on compliance factors conducted in the USA and Canada revealed that only age and political ideology were significantly associated with the preventative measures [13]. In Brazil’s general population, similarly as in our study, younger persons and males showed lower compliance with preventive measures during the COVID-19 pandemic [14]. Duong et al. observed that poor COVID-19 knowledge was positively associated with living alone among university students in Vietnam [47].

COVID-19 and adopting preventive measures, including vaccinations, quickly became a subject of political dispute [11]. Some political fractions even boycotted countries’ policies focusing on preventive measures. Furthermore, adherence to preventive measures became a form of political manifesto. In the Polish population of adult Internet users, lower adherence to preventive measures was found in persons not participating in the elections or supporting the extreme right-wing party when compared to the Law and Justice party, which won the parliamentary election. The role of political context was also mentioned by other authors [12,13,14]. Farias and Pilati confirmed, similarly as in Poland, that support for right-wing parties predicted non-compliance with preventive measures [14]. The earlier mentioned study by Farias and Pilati also showed that the effect of political partisanship on the support for COVID-19 preventive measures was moderated by conspiracy theories focusing on governmental malfeasance, personal wellbeing, and control of information [11]. A study performed in Italy showed that confidence in institutions was positively associated with political support for the central government and regional institutions. This, in turn, was associated with an increased likelihood of following the preventive measures [48]. It seems that the issue of confidence in governmental actions also appears in other studies. For example, Wang et al. reported for the USA and Canada that non-compliance factors also included, besides mixed messages from various sources and the inability to comply, distrust in the government [13]. Interestingly, compliance with preventive measures ordered by governors in the USA depended on the political leanings of the given counties [49].

Other authors have also shown a significant association between the use of social media and the adoption of preventive measures [46]. Furthermore, in this study, only selected potential predictors of adhering to COVID-19 preventive measures have been analyzed. However, some authors reported that non-adherence might be associated with risky health behaviors. For example, after analyzing the data from the SHARE COVID-19 Survey among a 50+ year-old population, Mendoza-Jimenez suggested that unfavorable health behaviors could be associated with lower adherence to selected preventive measures, e.g., hand-sanitizing or washing or covering coughs or sneezes [50]. In turn, Plohl and Musil reported that both COVID-19 risk perception and trust in science were independent predictors of compliance with preventive measures [9]. Alper et al. also reported risk perception as a significant positive predictor of adherence [15]. Other variables, including political conservatism, religious orthodoxy, conspiracy ideation and intellectual curiosity, were associated with compliance with such measures, but indirectly, with trust in science as a mediator.

### Limitations

The survey was performed with CAWI techniques, and the findings apply only to the population of adult Internet users. Therefore, they cannot be extrapolated to the whole adult population in Poland, especially to older adults and the elderly. Clearly, Internet use may be an important predictor of attitudes toward official recommendations to prevent the spreading of the new coronavirus.

Due to the limited volume of the questionnaire used in the survey, not all potential variables reported in the context of adherence to COVID-19 preventive measures have been included in the analysis. However, it is focused on the most tangible aspects in terms of implemented policies and undertaken health promotion interventions.

The study was based on a cross-sectional design that embodies the implicit assumption that the analyzed parameters remain unchanged over time and in the population. This is obviously a weakness of the approach, as it does not allow for consideration of the dynamics of social perceptions in a time of great epidemic challenge. Such a design also limits the possibility of using the results of survey data for assessing the causal relationships of actual processes in the populations.

The results reported in this paper originate from the quantitative approach and survey method. However, one should remember that the qualitative approach has also been used in many studies to assess the determinants of adherence to preventive measures. For example, analyzing social media content became a useful tool in this area [51,52,53,54].

## 5. Conclusions

The adjusted regression model showed many independent factors associated with compliance with COVID-19 preventive measures. The study, carried out among a large sample of Polish citizens, showed that among sociodemographic and economic factors, age, gender, marital status, vocational status and the level of household income play a role. Furthermore, there is a significant relationship between political sympathy or political indifference and lower compliance. To some degree, participation in religious practices may also be a significant predictor. As in other studies, firmer beliefs in conspiracy theories related to the COVID-19 pandemic are associated with lower compliance with preventive measures. Finally, both HL and eHL are positive predictors of adherence. It appears that the two latter factors are probably the most eligible for modification. Therefore, intense efforts to increase HL and eHL should be a target of pandemic strategies focused on the more positive reception of preventive measures.

## Figures and Tables

**Table 1:** Characteristics of the study group.

Variable	Response Options	%	n
Gender	Female	51.16	1233
	Male	48.84	1177
Education	Lower than secondary	20.5	494
	Secondary or post-sec. non-university	51.33	1237
	Bachelor’s degree	11.78	284
	Master’s degree	16.39	395
Place of residence	Rural	34.52	832
	Urban < 20,000	9.54	230
	Urban 20,000 to 100,000	23.45	565
	Urban 100,000 to 200,000	9.46	228
	Urban 200,000 to 500,000	9.21	222
	Urban > 500,000	13.82	333
Marital status	Married	49.46	1192
	Single	21.70	523
	Partnership	17.55	423
	Divorced, separated, or widowed	11.29	272
Vocational status	Public or private sector employee	49.5	1193
	Self-employed or farmer	8.17	197
	Retired or on disability pension	12.41	299
	High school or University student	10.92	263
	Vocationally passive incl. unemployed	19	458
Monthly net income per household member	≤1000 PLN	11.74	283
	1001–2000 PLN	40.46	975
	2001–3000 PLN	28.76	693
	>3000 PLN	19.04	459
Political support	Law and Justice	32.08	773
	Confederation	8.38	202
	Polish People’s Party	3.36	81
	Civic Coalition and allies	18.3	441
	Democratic Left Alliance	8.8	212
	Other ^a^	6.01	145
	Didn’t participate in the election	23.07	556
Participation in religious practices	Non-believer	13.24	319
	Non-practicing believer	32.49	783
	Less than 1 time per month	15.1	364
	1–3 times a month	19.5	470
	≥4 times a month	19.67	474
Time spent on social media daily	Not used	6.93	167
	Less than 30 min	23.82	574
	From 30 to less than 60 min	30.79	742
	From 60 to less than 120 min	21.50	518
	At least 120 min	16.97	409

^a^ Other = small parties or committees, invalid vote or not allowed to vote.

**Table 2:** Distribution to responses to items asking about the most popular COVID-19-related conspiracy beliefs.

Item	I Decidedly Do Not Agree	I Do Not Agree	I Rather Do Not Agree	Difficult to Say	I Rather Agree	I Agree	I Decidedly Agree
Coronavirus responsible for the pandemic is a result of genetic manipulations	5.93 (143)	7.76 (187)	7.51 (181)	39.00 (940)	18.51 (446)	10.29 (248)	11.00 (265)
Coronavirus was released from the laboratory on purpose	6.97 (168)	8.13 (196)	9.42 (227)	35.98 (867)	17.84 (430)	10.25 (247)	11.41 (275)
The risk related to the coronavirus is falsely exaggerated	13.28 (320)	14.61 (352)	12.24 (295)	22.74 (548)	15.23 (367)	9.00 (217)	12.9 (311)
The pandemic is a pretext for achieving hidden economic and political objectives	7.68 (185)	9.83 (237)	10.17 (245)	27.01 (651)	19.59 (472)	10.46 (252)	15.27 (368)
5G networks can spread the new coronavirus infection	31.58 (761)	14.69 (354)	11.49 (277)	29.05 (700)	6.14 (148)	3.61 (87)	3.44 (83)
In fact, there is no epidemic or pandemic being caused by the coronavirus	31.70 (764)	14.61 (352)	11.08 (267)	23.94 (577)	7.63 (184)	3.98 (96)	7.05 (170)

**Table 3:** Distribution of responses to items asking about adherence to COVID-19 preventive measures.

Item	Never % (n)	Rarely % (n)	Some-Times % (n)	Often % (n)	Very Often % (n)	Nearly Always % (n)	Always % (n)	Individual Score Mean (SD)
I keep a distance of at least 1–2 m from other people	1.83 (44)	4.69 (113)	9.09 (219)	14.02 (338)	13.44 (324)	30.91 (745)	26.02 (627)	5.29 (1.58)
I avoid touching my eyes, nose, and mouth	3.65 (88)	8.17 (197)	11.37 (274)	15.73 (379)	14.11 (340)	25.56 (616)	21.41 (516)	4.91 (1.74)
When coughing or sneezing, I cover my mouth and nose with a bent elbow or tissue	1.62 (39)	3.32 (80)	5.02 (121)	10.12 (244)	10.08 (243)	17.14 (413)	52.7 (1270)	5.86 (1.55)
I wear a mask correctly over my nose and mouth where it is mandatory or recommended	1.74 (42)	3.15 (76)	4.48 (108)	6.85 (165)	6.10 (147)	15.11 (364)	62.57 (1508)	6.08 (1.52)
I try to wash my hands as often as possible with soap and water or use antiviral hand sanitizer	0.62 (15)	1.87 (45)	5.93 (143)	11.29 (272)	12.49 (301)	20.29 (489)	47.51 (1145)	5.84 (1.42)
If possible, I avoid mass gatherings	3.24 (78)	5.77 (139)	8.26 (199)	12.94 (312)	14.81 (357)	22.7 (547)	32.28 (778)	5.28 (1.72)

**Table 4:** Univariable linear regression models for the preventive measures score.

Variable	Categories of Variable	B (Standard Error)	β	95% CI	*p*
HL		0.06 (0.007)	0.20	0.05–0.08	*p* < 0.001
eHL		0.05 (0.005)	0.18	0.04–0.06	*p* < 0.001
C19CBS		−0.23 (0.02)	−0.23	−0.27–−0.19	*p* < 0.001
Age		0.01 (0.002)	0.15	0.01–0.02	*p* < 0.001
Gender	Female ^#^				
	Male	−0.32 (0.05)	−0.13	−0.41–−0.22	*p* < 0.001
Marital status	Married ^#^				
	Single	−0.24 (0.06)	−0.08	−0.37–−0.12	*p* < 0.001
	Partnership	−0.18 (0.07)	−0.06	−0.32–−0.04	0.01
	Divorced, separated or widowed	0.12 (0.08)	0.03	−0.04–0.28	0.15
Vocational status	Public or private sector employee ^#^				
	Self-employed or farmer	0.07 (0.09)	0.02	−0.11–0.26	0.45
	Retired or on disability pension	0.55 (0.08)	0.15	0.40–0.71	*p* < 0.001
	High school or university student	−0.08 (0.08)	−0.02	−0.24–0.09	0.35
	Vocationally passive incl. unemployed	0.20 (0.07)	0.06	0.07–0.33	0.003
Monthly net income per household member	2001–3000 PLN ^#^				
	≤1000 PLN	−0.11 (0.08)	−0.03	−0.28–0.05	0.17
	1001–2000 PLN	−0.04 (0.06)	−0.01	−0.16–0.08	0.56
	>3000 PLN	−0.16 (0.07)	−0.05	−0.30–−0.03	0.02
Political support	Law and Justice ^#^				
	Confederation	−0.71 (0.1)	−0.16	−0.9–−0.52	*p* < 0.001
	Polish People’s Party	0.11 (0.14)	0.02	−0.17–0.39	0.43
	Civic Coalition and allies	−0.08 (0.07)	−0.03	−0.23–0.06	0.25
	Democratic Left Alliance	−0.11 (0.09)	−0.02	−0.29–0.08	0.25
	Other ^a^	−0.44 (0.11)	−0.08	−0.65–−0.22	*p* < 0.001
	Didn’t participate in the election	−0.31 (0.07)	−0.11	−0.44–−0.18	*p* < 0.001
Participation in religious practices	Non-practicing believer ^#^				
	Non-believer	−0.15 (0.08)	−0.04	−0.31–0.01	0.06
	Less than 1 time per month	−0.03 (0.08)	−0.01	−0.19–0.12	0.66
	1–3 times a month	0.01 (0.07)	0.003	−0.13–0.15	0.89
	≥4 times a month	0.15 (0.07)	0.05	0.01–0.29	0.03

Abbreviations: HL—health literacy, eHL—e-health literacy, C19CBS—COVID-19-related conspiracy beliefs score. ^a^ Other—small parties or committees, invalid vote or not allowed to vote, ^#^—referential category of the variable.

**Table 5:** Multivariable linear regression model of the preventive measures score.

Variable	Categories of Variable	B (Standard Error)	β	95% CI	*p*
HL		0.04 (0.01)	0.13	0.03–0.06	<0.001
eHL		0.03 (0.01)	0.10	0.02–0.04	<0.001
C19CBS		−0.24 (0.02)	−0.24	−0.28–−0.20	<0.001
Age		0.01 (0.003)	0.07	0.001–0.01	0.03
Gender	Female ^#^				
	Male	−0.35 (0.05)	−0.15	−0.45–−0.25	<0.001
Marital status	Married ^#^				
	Single	−0.05 (0.07)	−0.02	−0.19–0.10	0.51
	Partnership	0.01 (0.07)	0.002	−0.13–0.15	0.92
	Divorced, separated or widowed	−0.003 (0.08)	−0.001	−0.17–0.16	0.97
Vocational status	Public or private sector employee ^#^				
	Self-employed or farmer	0.12 (0.09)	0.03	−0.06–0.31	0.18
	Retired or on disability pension	0.39 (0.10)	0.10	0.20–0.57	*p* < 0.001
	High school or university student	−0.001 (0.10)	0.0002	−0.19–0.19	0.99
	Vocationally passive incl. unemployed	0.26 (0.07)	0.08	0.12–0.39	*p* < 0.001
Monthly net income per household member	2001–3000 PLN ^#^				
	≤1000 PLN	−0.06 (0.09)	−0.01	−0.23–0.12	0.52
	1001–2000 PLN	0.003 (0.06)	0.001	−0.11–0.12	0.96
	>3000 PLN	−0.14 (0.07)	−0.05	−0.28–−0.01	0.04
Political support	Law and Justice ^#^				
	Confederation	−0.32 (0.10)	−0.07	−0.51–−0.13	0.001
	Polish People’s Party	0.17 (0.13)	0.03	−0.10–0.43	0.21
	Civic Coalition and allies	−0.03 (0.07)	−0.01	−0.18–0.11	0.66
	Democratic Left Alliance	−0.13 (0.10)	−0.03	−0.32–0.06	0.19
	Other ^a^	−0.18 (0.11)	−0.04	−0.4–0.04	0.11
	Didn’t participate in the election	−0.19 (0.07)	−0.06	−0.32–−0.05	0.006
Participation in religious practices	Non-practicing believer ^#^				
	Non-believer	−0.24 (0.08)	−0.07	−0.4–−0.08	0.003
	Less than 1 time per month	−0.08 (0.08)	−0.02	−0.23–0.07	0.27
	1–3 times a month	−0.02 (0.07)	−0.01	−0.16–0.12	0.77
	≥4 times a month	0.07 (0.07)	0.02	−0.07–0.21	0.35

Abbreviations: HL—health literacy, eHL—e-health literacy, C19CBS—COVID-19-related conspiracy beliefs score. ^a^ Other—small parties or committees, invalid vote or not allowed to vote, ^#^—referential category of the variable.

## Data Availability

The data are not publicly available due to privacy and ethical restrictions. The authors did not include in the information about the study provided to the participants that the public access to the data obtained during the survey may be considered. Access to the data will be granted on a case-by-case basis on a justified request after receiving consent from the Bioethical Committee at Jagiellonian University.

## Supplementary Materials

The following supporting information can be downloaded at: https://www.mdpi.com/article/10.3390/ijerph19148346/s1, Supplementary Material File S1: The items from the survey questionnaire that were used in the analysis reported in the paper.
