# Supplementary material for: The Roles of Health and e-Health Literacy, Conspiracy Beliefs and Political Sympathy in the Adherence to Preventive Measures Recommended during the Pandemic
Source: Int J Environ Res Public Health. 2022 Jul 8;19(14):8346. doi: 10.3390/ijerph19148346 (PMC9321506; doi:10.3390/ijerph19148346)
Supplement: Supplementary file 1 [file ijerph-19-08346-s001.zip › ijerph-1790437-supplementary.pdf]

Supplementary material File S1 – The items from the survey questionnaire that were used in the analysis reported in the paper

Kwestionariusz ankiety

CZĘŚĆ A.

Zaznaczając odpowiednią kratkę, proszę odpowiedzieć na pytanie: **JAK TRUDNE SĄ DLA PANA/PANI POSZCZEGOLNE CZYNNOŚCI** opisane poniżej:

Pytanie	bardzo łatwe	łatwe	trudne	niemoż-liwe	trudno powiedzieć /nie dotyczy
…znajdowanie informacji na temat leczenia chorób, które u PANA/I występują?	☐	☐	☐	☐	☐
…dowiedzenie się, gdzie można uzyskać fachową pomoc, kiedy PAN/I źle się czuje?	☐	☐	☐	☐	☐
…rozumienie co mówi do PANA/I lekarz?	☐	☐	☐	☐	☐
…zrozumienie zaleceń lekarza lub farmaceuty na temat sposobu przyjmowania przepisanego leku?	☐	☐	☐	☐	☐
…ocenienie, kiedy potrzebuje PANA/I zasięgnąć opinii innego lekarza?	☐	☐	☐	☐	☐
…wykorzystanie informacji uzyskanych od lekarza w trakcie podejmowania decyzji dotyczących PANA/I choroby?	☐	☐	☐	☐	☐
…stosowanie się do zaleceń lekarza lub farmaceuty?	☐	☐	☐	☐	☐
…znajdowanie informacji dotyczących radzenia sobie ze stresem, depresją czy innymi podobnymi stanami?	☐	☐	☐	☐	☐
…zrozumienie ostrzeżeń dotyczących zagrożeń zdrowotnych związanych z takimi zachowaniami jak palenie, niska aktywność fizyczna czy nadużywanie alkoholu?	☐	☐	☐	☐	☐
…zrozumienie, dlaczego powinien PAN/I poddawać się badaniom przesiewowym (masowym, bezpłatnym)?	☐	☐	☐	☐	☐
…ocenienie, czy wiarygodne są znajdowane w mediach informacje o czynnikach zagrażających zdrowiu?	☐	☐	☐	☐	☐
…zdecydowanie, w oparciu o informacje z mediów, jak bronić się przed chorobą?	☐	☐	☐	☐	☐
…znajdowanie informacji na temat zachowań, które są korzystne dla stanu psychicznego?	☐	☐	☐	☐	☐
…rozumienie porad dotyczących zdrowia otrzymywanych od członków rodziny i przyjaciół?	☐	☐	☐	☐	☐
…rozumienie informacji z mediów na temat sposobów poprawy zdrowia?	☐	☐	☐	☐	☐
…ocenienie, które z codziennych zachowań wpływają na PANA/I zdrowie?	☐	☐	☐	☐	☐

CZĘŚĆ B.

Chcielibyśmy teraz zapytać o Pana/Pani opinię i doświadczenia dotyczące wykorzystania Internetu w odniesieniu do informacji dotyczących zdrowia. W przypadku każdego stwierdzenia, proszę wskazać odpowiedź, która najlepiej pokazuje Pana/Pani opinię i doświadczenia w tym momencie.

1. Jakie są Pana/Pani odczucia co do przydatności Internetu w podejmowaniu decyzji dotyczących swojego zdrowia?
   a. Zupełnie nie jest przydatny
   b. Nie jest przydatny
   c. Nie jestem pewien/pewna
   d. Jest przydatny
   e. Jest bardzo przydatny
2. Jak ważny jest dla Pana/Pani dostęp do zasobów dotyczących zdrowia w Internecie?
   a. Zupełnie nie jest ważny
   b. Nie jest ważny
   c. Nie jestem pewien/pewna
   d. Jest ważny
   e. Jest bardzo ważny
3. (eHEALS1) Wiem jakie zasoby dotyczące zdrowia są dostępne w Internecie?
   a. Zdecydowanie się nie zgadzam
   b. Nie zgadzam się
   c. Nie mam zdania
   d. Zgadzam się
   e. Zdecydowanie się zgadzam
4. (eHEALS2) Wiem gdzie znaleźć pomocne zasoby dotyczące zdrowia w Internecie.
   a. Zdecydowanie się nie zgadzam
   b. Nie zgadzam się
   c. Nie mam zdania
   d. Zgadzam się
   e. Zdecydowanie się zgadzam
5. (eHEALS3) Wiem jak odszukać pomocne zasoby dotyczące zdrowia w Internecie.
   a. Zdecydowanie się nie zgadzam
   b. Nie zgadzam się
   c. Nie mam zdania
   d. Zgadzam się
   e. Zdecydowanie się zgadzam
6. (eHEALS4) Wiem jak korzystać z Internetu aby odpowiedzieć na swoje pytania dotyczące zdrowia
   a. Zdecydowanie się nie zgadzam
   b. Nie zgadzam się
   c. Nie mam zdania
   d. Zgadzam się
   e. Zdecydowanie się zgadzam
7. (eHEALS5) Wiem jak wykorzystać informacje dotyczące zdrowia, które znajduję w Internecie, aby sobie pomóc.
   a. Zdecydowanie się nie zgadzam
   b. Nie zgadzam się
   c. Nie mam zdania
   d. Zgadzam się
   e. Zdecydowanie się zgadzam
8. (eHEALS6) Mam umiejętności potrzebne do oceny zasobów dotyczących zdrowia, które znajduję w Internecie
   a. Zdecydowanie się nie zgadzam
   b. Nie zgadzam się
   c. Nie mam zdania
   d. Zgadzam się
   e. Zdecydowanie się zgadzam
9. (eHEALS7) Potrafię odróżnić zasoby dotyczące zdrowia dostępne w Internecie o wysokiej jakości od tych o niskiej jakości
   a. Zdecydowanie się nie zgadzam
   b. Nie zgadzam się
   c. Nie mam zdania
   d. Zgadzam się
   e. Zdecydowanie się zgadzam
10. (eHEALS8) Czuję się pewnie gdy wykorzystuję informacje z Internetu do podejmowania decyzji dotyczących zdrowia.
   a. Zdecydowanie się nie zgadzam
   b. Nie zgadzam się
   c. Nie mam zdania
   d. Zgadzam się
   e. Zdecydowanie się zgadzam

CZĘŚĆ C.

Proszę odpowiedzieć w jakim stopniu stara się Pan/Pani przestrzegać zaleceń dotyczących zapobiegania zakażeniu koronawirusem. W przypadku każdego pytania, proszę wskazać odpowiedź, która najlepiej oddaje Pana/Pani działania.

	Zawsze	Prawie zawsze	Bardzo często	Często	Czasami	Rzadko	Nigdy
Zachowuję odległość co najmniej 1-2 metrów między sobą a innymi ludźmi	☐	☐	☐	☐	☐	☐	☐
Unikam dotykania oczu, nosa i ust	☐	☐	☐	☐	☐	☐	☐
Podczas kaszlu i kichania zakrywam usta i nos zgiętym łokciem lub chusteczką	☐	☐	☐	☐	☐	☐	☐
Noszę maseczkę założoną poprawnie na nos i usta w miejscach, w których jest to obowiązkowe lub zalecane	☐	☐	☐	☐	☐	☐	☐
Staram się jak najczęściej myć ręce wodą i mydłem lub używam przeciwwirusowego środka odkażającego	☐	☐	☐	☐	☐	☐	☐
Jeśli to możliwe, unikam miejsc, w których gromadzą się ludzie.	☐	☐	☐	☐	☐	☐	☐

CZĘŚĆ E.

Proszę wybrać odpowiedź która najlepiej odpowiada Pana/Pani przekonaniom i poglądom

	zdecydo-wanie się nie zgadzam	nie zgadzam się	raczej się nie zgadzam	trudno powiedzieć	raczej się zgadzam	nie zgadzam się	zdecydo-wanie się zgadzam
47. Koronawirus odpowiedzialny za pandemię jest wynikiem manipulacji genetycznych przeprowadzonych przez człowieka	☐	☐	☐	☐	☐	☐	☐
48. Koronawirus został celowo uwolniony z laboratorium	☐	☐	☐	☐	☐	☐	☐
49. Zagrożenie związane z koronawirusem jest sztucznie wyolbrzymiane	☐	☐	☐	☐	☐	☐	☐
50. Pandemia jest pretekstem do osiągania ukrytych celów ekonomicznych i politycznych	☐	☐	☐	☐	☐	☐	☐
51. Sieć 5G może mieć związek z szerzeniem się zakażenia nowym koronawirusem	☐	☐	☐	☐	☐	☐	☐
52. W rzeczywistości nie ma epidemii ani pandemii koronawirusa	☐	☐	☐	☐	☐	☐	☐

Metryczka

1. Płeć: mężczyzna…. kobieta ….
2. Wiek w latach ....
3. Miejsce zamieszkania
    1. wieś
    2. miasta o liczbie mieszkańców poniżej 20 tys.
    3. miasta o liczbie mieszkańców 20-100 tys.
    4. miasta o liczbie mieszkańców 100-200 tys.
    5. miasta o liczbie mieszkańców 200-500 tys.
    6. miasta o liczbie mieszkańców 500 tys. i więcej
4. Wykształcenie
    1. bez wykształcenia
    2. podstawowe ukończone
    3. gimnazjalne
    4. zasadnicze zawodowe
    5. średnie ogólnokształcące lub zawodowe
    6. pomaturalne
    7. wyższe ze stopniem inżyniera lub licencjata
    8. wyższe ze stopniem magistra lub równorzędnym
5. Dochód netto przypadający na domownika w gospodarstwie domowym w zł:
    1. nie więcej niż 500 zł
    2. 501 – 1000 zł
    3. 1001 – 1500 zł
    4. 1501 – 2000 zł
    5. 2001 – 3000 zł
    6. 3001 - 4000
    7. powyżej 4000 zł
6. Status zawodowy
    1. pracownik sektora publicznego lub prywatnego
    2. praca dorywcza
    3. prywatny przedsiębiorca
    4. rolnik
    5. rencista
    6. emeryt
    7. uczeń
    8. student
    9. bezrobotny
    10. bierny zawodowo z innych przyczyn
    11. inny
7. Stan cywilny (wg kategorii
    1. panna/kawaler
    2. żonaty/zamężna
    3. wdowa/wdowiec
    4. rozwiedziony/a
    5. w separacji
    6. związek partnerski
8. Na jaką partię głosował/a Pan/Pani w wyborach do Sejmu w 2019 roku:
    a. Prawo i Sprawiedliwość
    b. Konfederacja Wolność i Niepodległość
    c. Komitet Wyborczy Prawica
    d. Polskie Stronnictwo Ludowe
    e. Koalicja Obywatelska PO .N IPL Zieloni
    f. Sojusz Lewicy Demokratycznej

g. Akcja Zawiedzionych Emerytów i Rencistów
h. Koalicja Bezpartyjni i Samorządowcy
i. Skuteczni Piotra Liroya-Marca
j. Komitet Wyborczy Wyborców Mniejszość Niemiecka
k. oddałem/am głos nieważny
l. nie mogłem/am jeszcze głosować
m. nie wziąłem/am udziału w tych wyborach

9. Jak często przeciętnie w ciągu miesiąca bierze Pan/Pani udział w nabożeństwach lub innych spotkaniach o charakterze religijnym?

   a. jestem niewierzący/a
   b. jestem wierzący/a ale nie praktykuję
   c. rzadziej niż 1 w miesiącu
   d. 1 raz w miesiącu
   e. 2-3 razy w miesiącu
   f. 4 razy w miesiącu
   g. 5 razy w miesiącu lub częściej
